# The Interplay between Nitrosative Stress, Inflammation, and Antioxidant Defense in Patients with Lichen Planus

**DOI:** 10.3390/antiox13060670

**Published:** 2024-05-30

**Authors:** Mircea Tampa, Ilinca Nicolae, Corina Daniela Ene, Cristina Iulia Mitran, Madalina Irina Mitran, Clara Matei, Simona Roxana Georgescu

**Affiliations:** 1 Department of Dermatology, ‘Carol Davila’ University of Medicine and Pharmacy, 020021 Bucharest, Romania; dermatology.mt@gmail.com (M.T.); srg.dermatology@gmail.com (S.R.G.); 2 Department of Dermatology, ‘Victor Babes’ Clinical Hospital for Infectious Diseases, 030303 Bucharest, Romania; drnicolaei@yahoo.ro; 3 Departments of Nephrology, ‘Carol Davila’ University of Medicine and Pharmacy, 020021 Bucharest, Romania; 4 Department of Nephrology, ‘Carol Davila’ Nephrology Hospital, 010731 Bucharest, Romania; 5 Department of Microbiology, ‘Carol Davila’ University of Medicine and Pharmacy, 020021 Bucharest, Romania; cristina.iulia.mitran@gmail.com (C.I.M.); madalina.irina.mitran@gmail.com (M.I.M.)

**Keywords:** lichen planus, nitrosative stress, inflammation, antioxidants, markers

## Abstract

Background: Lichen planus (LP) is a chronic inflammatory skin disease of unelucidated etiology. LP immunopathogenesis is mainly governed by cytotoxic T lymphocytes that mediate an immune response in basal keratinocytes, which may transform into a reservoir of antigens able to initiate an autoimmune reaction. However, other pathogenic pathways complement these mechanisms. Recent studies highlight the involvement of nitrosative stress in the pathogenesis of chronic inflammatory skin diseases. Current data on its role in the pathogenesis of LP are scarce. Methods: In this article, we investigated nitrosative stress in 40 cutaneous LP (CLP) patients compared to 40 healthy subjects using serum markers including nitrosative stress markers—direct nitrite, total nitrite, nitrate and symmetric dimethylarginine (SDMA), total antioxidant status (TAS), and hsCRP, a marker of inflammation, and analyzed the relationship between nitrosative stress, antioxidant defense, and inflammation to offer new insights into the role of the NO pathway in LP pathogenesis. Results: We identified significantly higher serum levels of direct nitrite, total nitrite, nitrate, SDMA and hsCRP, and significantly lower levels of TAS in CLP patients versus controls. There were significant negative correlations between the serum levels of TAS and significantl positive correlations between the serum levels of hsCRP and the analyzed nitrosative stress markers in patients with CLP. Conclusion: Our results indicate an increased level of nitrosative stress in LP patients that correlates with a pro-inflammatory status and altered antioxidant defense.

## 1. Introduction

Chronic inflammatory skin diseases represent a heterogeneous group of conditions characterized by an abnormal reaction of immune cells to a variety of internal or external triggers, leading to the onset and persistence of an inflammatory process, typically progressing into a chronic state [1]. Lichen planus (LP) remains a condition of unknown etiology, although, in terms of pathogenesis, most studies attribute a role to T cells, and subsequently it is considered a T-cell-mediated autoimmune disease. Current evidence suggests that an imbalance of the cellular immune response occurs, induced by exogenous or endogenous factors, leading to the development of an immune response against self-antigens governed especially by CD8+ T cells [2,3]. It is worth mentioning that the inflammatory infiltrate encountered in LP lesions is mostly composed of CD8+ T cells. Along with CD8+ T cells, apoptotic keratinocytes are identified. The main mechanisms involved in keratinocyte apoptosis are the secretion of tumor necrosis factor (TNF) that binds to the keratinocyte receptor, the release of granzymes and the interaction between CD85L on the lymphocyte surface and CD95 on the keratinocyte surface. It is assumed that the antigen is either a self-peptide or a heat shock protein. Under inflammatory conditions, keratinocytes have been shown to express heat shock proteins [4,5,6,7].

Regarding LP pathogenesis, two hypotheses have been proposed, namely the “chance encounter” hypothesis and the “direct migration hypothesis”. The first one refers to the immune surveillance performed by CD8+ T cells that encounter the keratinocyte antigen by chance in the epithelium. Arguments in favor of this hypothesis are the presence of CD8+ T cells in the oral epithelium in healthy people and the late onset of the disease, conditioned by the moment of encounter between lymphocyte and the keratinocyte antigen. The “directed migration” hypothesis suggests that chemokines produced by keratinocytes attract both antigen-specific T cells and non-specific T cells to the lesional tissue. It was found that considerable amounts of T cells in LP lesions are non-clonal, and not all CD8+ T-cell clones in the lesion exhibit cytotoxic activity against autologous lesional keratinocytes in laboratory settings [5].

In autoimmune diseases, elevated levels of reactive oxygen species (ROS) and reactive nitrogen species (RNS) have been identified, demonstrating the close relationship between the immune system and oxidative stress. ROS regulate the relationship between innate and adaptive immune cells and mediate the antigen-presenting process that promotes T-cell activation [8]. Inflammatory cells can release various ROS/RNS that exert harmful effects on tissues, leading to the development of oxidative/nitrosative stress. Nitrosative/oxidative stress represents an important source of markers that can be used in the diagnosis and assessment of response to therapy in dermatological conditions [1,9,10].

Considering that the skin is the body’s interface with the external environment, and in the environment, there are numerous aggressors that can create an imbalance between oxidants and antioxidants, it is considered that oxidative stress plays an important role in chronic inflammatory skin diseases. Regarding LP, there are numerous studies that investigate the level of oxidative stress in patients with oral LP [11,12,13], the studies being less numerous in the case of cutaneous LP. Recently, we performed a review that focused on oxidative stress markers in cutaneous LP (CLP) to provide a more complete understanding of the involvement of oxidative stress in this disease and identified that there is an increased level of prooxidants in conjunction with a decrease in the activity of enzymatic and non-enzymatic antioxidants. In addition, in some studies, there is a positive correlation between the levels of oxidative stress markers and disease duration [14].

An insufficiently unexplored direction in CLP pathogenesis is the nitric oxide (NO) pathway. Nitrosative stress is the result of the excessive generation of nitrogen-based free radicals including nitric oxide and nitrogen dioxide [15]. RNS are distinct NO-derived molecules with a high reactivity considering that in their structure, there are unpaired electrons. When RNS are produced in excess, the cellular redox balance is altered, resulting in nitrosative stress, which is similar to oxidative stress. RNS exert harmful effects on cell components, proteins, lipids, and nucleic acids that mainly include tyrosine nitration, lipid peroxidation, and DNA strand breaks, leading to the alteration of the cell membrane, the impairment of the enzyme function, and the activation of signaling pathways involved in cell death [16].

NO is a free radical that displays many functions in the human body such as the modulation of blood pressure, neuronal communication, anti-infectious defense, wound healing, etc. It is synthesized via a chemical reaction catalyzed by NOS from the nitrogen residue of the amino acid L-arginine in the presence of nicotinamide adenine dinucleotide phosphate and molecular oxygen [17]. The significance of NO in both the cell homeostasis and diseases of the skin has garnered increasing attention in research, especially since the discovery two decades ago that nearly all skin cell types have the ability to produce this redox signaling molecule. It is now clear that NO plays a crucial role in various physiological processes of the skin [keratinocyte and fibroblast proliferation, collagen synthesis, microcirculation, innervation, immune response]; however, disruptions in NO signaling have significant implications for skin health [18]. The metabolites involved in the NO pathway have been extensively studied in relation to the development and progression of various diseases, but data on skin diseases are scarce. Despite the significant advancements made in recent years, understanding the pathogenic mechanisms that are involved in LP remains a complex challenge in contemporary medicine. NO may promote the proliferation of T cells in LP lesions, therefore NO can be regarded as a molecule that mediates the proliferation and differentiation of T cells [19]. Increased levels of NO lead to keratinocyte, fibroblast, and epithelial cell damage. It has been hypothesized that NO may lead to erosive or ulcerative lesions, which are present in oral LP [20].

In this article, we investigated nitrosative stress in CLP patients versus a control group using serum markers including nitrosative stress markers—direct nitrite, total nitrite, nitrate and symmetric dimethylarginine (SDMA), total antioxidant status (TAS) and inflammation markers (high-sensitivity C-reactive protein—hsCRP, orosomucoid and erythrocyte sedimentation rate—ESR)—and analyzed the relationship between nitrosative stress, antioxidant defense, and inflammation to offer new insights into the role of the NO pathway in LP pathogenesis.

## 2. Materials and Methods

### 2.1. Study Participants

**Figure 1:**
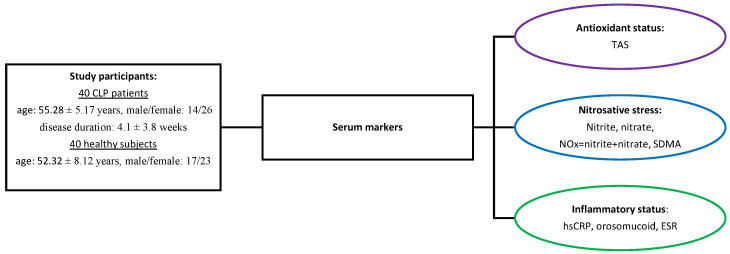
Study design.

We conducted an observational study in the Dermatology Clinic of the Clinical Hospital “Dr. Victor Babeș”. In the study, we included 40 patients with LP and 40 age- and sex-matched controls, healthy subjects who presented with conditions such as skin tags or nevi, afflictions that do not influence the serum levels of the studied markers (Figure 1). The diagnosis of LP was assigned based on the clinical aspects and the histopathological examination. The clinical forms of LP were as follows: classic LP—31 patients, hypertrophic LP—6 patients, annular LP—3 patients. Six of them had oral lesions. To evaluate the extension of the disease, we calculated the body surface area (BSA) affected by LP: 0–30%—23 patients, 31–60%—15 patients, >60%—2 patients.

All study participants signed an informed consent form, agreeing to participate.

The study received the approval of the Ethics Committee of the Dr. Victor Babes Hospital, Bucharest (no. 2/18.09.2017). The inclusion and exclusion criteria of study participants are presented in Table 1.

### 2.2. Sample Collection

Venous blood samples were collected a jeun, using the holder vacutainer system. An amount of 7 mL of blood was taken. The samples were centrifuged for 10 min at 6000× *g*. Inappropriate samples (hemolyzed or lactescent) were excluded. The samples were immediately processed or stored at a low temperature (−80 °C).

### 2.3. Laboratory Determinations

**Figure 2:**
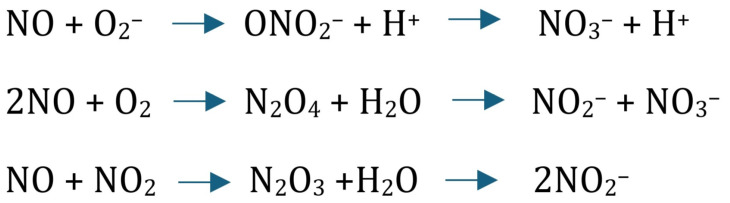
Nitrites and nitrates are the end products of NO in vivo. Therefore, the sum of the 2 compounds is the most reliable indicator of total NO production.

We performed all determinations according to manufacturer instructions.

a. Determination of Nitrate (NO_3_^−^) and Nitrite (NO_2_^−^). This determination reflects the level of NO (Figure 2).

We determined the levels of nitrate and nitrite in the serum of study participants through a colorimetric assay (the Griess method) [21]. The nitrate/nitrite colorimetric assay (CAYMAN CHEMICAL CAY 780001, Ann Arbor, MI, USA) is a precise and simple method for determination of total and individual nitrate and nitrite levels. The first step of the process is based on the conversion of nitrate to nitrite under the action of nitrate reductase. Subsequently, we added the Griess reagents, which are aimed to convert nitrite into a deep purple azo compound, into the reaction. Given that the azo compound is a chromophore, we carried out the photometric measurement of the absorbance, which is a precise method for the determination of NO_2_^−^ concentration. The colorimetric measurement was made at a wavelength of 540 nm, and a Tecan [GmbH, Grodig, Austria] analyzer was used. The inter-assay and intra-assay coefficients of variation were 3.4% and 2.7%, respectively. The results are expressed as µmol/L.

We determined:

- direct nitrite;
- total nitrite;
- nitrate [it is calculated by the difference between total nitrites and direct nitrites].

b. Determination of SDMA

SDMA was determined by the ELISA method [22] (MyBiosource, San Diego, CA, USA, MBS167761). The plate was pre-coated with human SDMA antibodies [primary antibodies]. The samples were added, and SDMA present in the samples was bound to antibodies coated on the wells. Subsequently, the second human SDMA antibodies were added and bound to the SDMA in the sample. The enzyme used was streptavidin-HRP. Color intensity was directly proportional to the concentration of SDMA in the sample. For the evaluation of the final product, a semi-automatic analyzer was employed with a wavelength of 450 nm (Tecan GmbH, Grodig, Austria). The unit of measurement was µmol/L.

c. Determination of TAS

TAS determination was performed using the ELISA method [23] (MyBiosource, San Diego, CA, USA, MBS9304157). The method employed anti-TAS monoclonal antibodies and enzyme-labelled TAS. An HRP colorimetric detection system was used. The final result was read at a wavelength of 450 nm. Since TAS in the sample and enzyme-labelled TAS compete for binding to the same antibody, the color intensity is inversely proportional to the amount of TAS in the sample. For the evaluation of the final product, a semi-automatic analyzer was employed with a wavelength 450 nm (Tecan GmbH, Grodig, Austria). The unit of measurement was µmol/L.

d. Determination of hsCRP

The serum level of hsCRP was determined by the latex immunoturbidimetric method. The principle of the method is based on an antigen–antibody agglutination reaction, between hsCRP in the sample and anti-CRP antibodies coupled with latex particles. The result is read at a wavelength of 570 nm (HumaStar300, Weisbaden, Germany). The change in absorbance is directly proportional to the concentration of hsCRP in the sample. Results are expressed in mg/dL.

e. Determination of orosomucoid

The levels of orosomucoid were determined through an immunonephelometric assay (MyBiosource, San Diego, CA, USA MBS901995 kit). The results were read at a wavelength of 340 nm using a HumanStar300 analyzer (Weisbaden, Germany). The unit of measurement was g/L.

### 2.4. Statistical Analysis

We used Wilcoxon test to compare the two groups (patients with CLP and controls). The relationship between pairs of two parameters was evaluated by Spearman’s correlation coefficient according to the results of the analysis performed with Kolmogorov–Smirnov test regarding the normality of data. A *p* value < 0.05 was considered significant.

## 3. Results

We identified higher levels of hsCRP and orosomucoid and lower levels of TAS in CLP patients compared to controls. There were no significant differences between groups regarding the levels of ESR (Table 2).

The mean serum levels of the nitrosative stress markers (direct nitrite, total nitrite, nitrate, and SDMA) were significantly higher in patients with CLP compared to healthy controls (Table 3).

**Figure 3:**
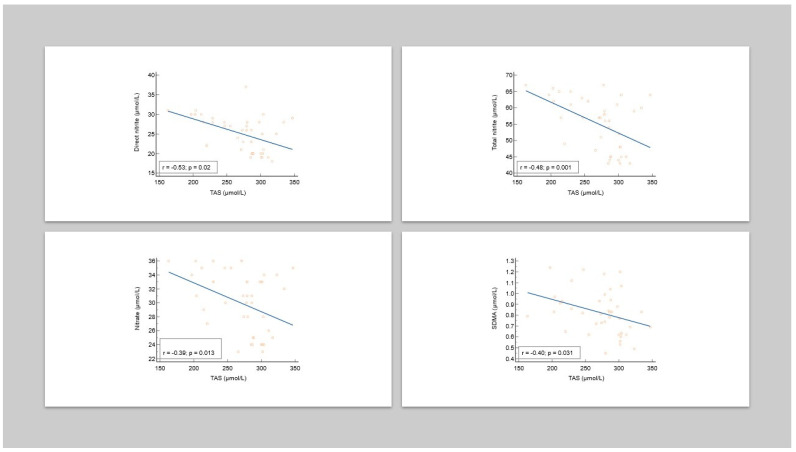
Correlations between TAS and nitrosative stress markers in CLP patients.

There were significantly negative correlations between the serum levels of TAS and the analyzed nitrosative stress markers (direct nitrite, total nitrite, nitrate, and SDMA) in patients with CLP (Table 4, Figure 3).

**Figure 4:**
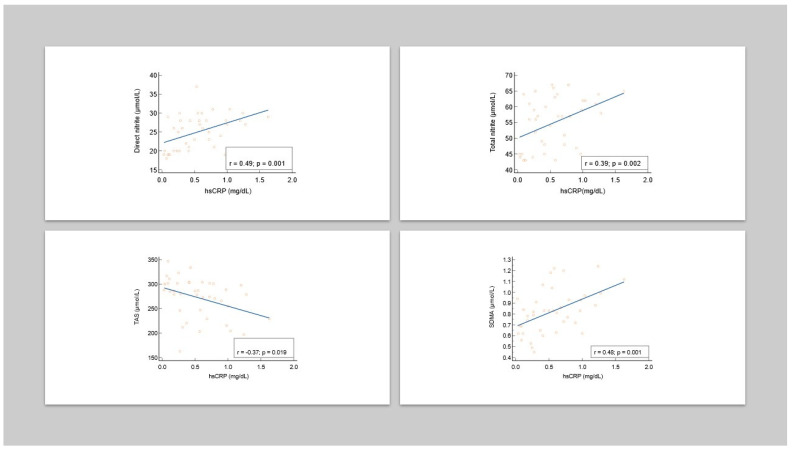
Correlations between hsCRP and nitrosative stress markers in CLP patients.

In addition, we analysed the relationship between inflammation and nitrosative stress (Table 5). Among the studied markers of inflammation, we chose hsCRP as a marker given that we found the most significant results. There were significantly positive correlations between the serum levels of hsCRP and the analyzed nitrosative stress markers (direct nitrite, total nitrite, and SDMA) in patients with CLP. There was a lack of correlation between the serum levels of hsCRP and nitrate in patients with CLP (Figure 4). In addition, there was a negative correlation between TAS and hsCRP (Table 5).

There were no statistically significant correlations between disease characteristics and the studied markers, except for the positive correlation between serum nitrate levels and BSA (Table 6).

## 4. Discussion

NO can be considered a “double-edged sword.” Low levels of NO are involved in cell homeostasis, while high levels of NO can disturb the capacity of cells to destroy microorganisms and malignant cells harboring mutations. Subsequently, NO regulates inflammatory and immunological responses [24]. NO is an important player in the physiological processes that take place in the skin, participating in the barrier function and antimicrobial defense, melanogenesis, and the skin response to ultraviolet radiation [25].

Numerous markers are available to evaluate nitrosative/oxidative stress, but none of these can be regarded as the ideal marker [26]. There are several important gaps in the study of nitrosative/oxidative stress in CLP. Studies evaluating nitrosative/oxidative stress in patients with CLP are limited, and most of them include a small number of patients [14,27]. Our study brings to attention the role of nitrosative stress in CLP and its interconnection with inflammation, two processes that influence each other. The involvement of oxidative processes and the consequences of RNS/ROS accumulation are not fully understood in LP. Cellular lesions caused by oxidative stress can exacerbate the autoimmune process in LP, creating favorable conditions for autoantigen generation that induce immune response activation and formation of autoantibodies. Under oxidative stress conditions, by-products of lipid peroxidation such as malondialdehyde (MDA) and 4-hidroxynonenal (4-HNE) can form protein adducts with immunogenic properties [28,29].

The NO precursors that are found in the skin, including nitrites, nitrates and S-nitrosothiols [RSNOs] constitute an important reservoir of NO [30]. Systemic circulating nitrate originates from two main pathways, diet and endogenous NO oxidation. Endogenously, nitrate is converted to nitrite; therefore, approximately 93% of nitrite results from nitrate. Most of the circulating nitrite is converted to NO [31]. Considering these processes, in this study, we determined serum nitrate and nitrite levels in CLP patients, which are actually indicators of NO homeostasis. An imbalance in NO homeostasis can underlie the appearance of skin pathologies.

When RNS are produced in excess, the antioxidant capacity is overwhelmed and the cellular function is disrupted, leading to apoptosis, a phenomenon observed in LP lesions [17]. The role of NO has been demonstrated in several autoimmune skin conditions such as vitiligo or alopecia areata. The important autoimmune component involved in the pathogenesis of CLP should be considered. Thus, a recent study by Taskin et al. highlighted increased levels of NO, ONOO^−^, and NOS in patients with alopecia areata compared to the control group and hypothesized that NOS activity may represent a biomarker for predicting alopecia areata [32]. Similarly, Mulayim et al. identified increased levels of NO and nitrotyrosine in patients with vitiligo compared to the control group, demonstrating the role of nitrosative stress in the pathogenesis of this condition [33].

In this study, we identified significantly higher serum nitrite and nitrate levels in CLP patients compared to controls, which pathophysiologically means elevated NO levels. There are few studies in the literature that focus on the relationship between NO and CLP. Sezer et al. identified increased levels of NO and lipid peroxidation in CLP patients compared to healthy individuals [34]. These results are in concordance with those obtained by Hassan et al. [35]. Similar results were obtained by Aly et al. who additionally investigated whether there are correlations between clinical forms of CLP and markers of oxidative stress, concluding that there is no correlation in this regard. Interesting, they identified a positive correlation between the duration of the disease and NO levels [36]. In the previously mentioned studies, the NO determinations were carried out in the serum of patients. Abdel Karim et al. assessed the levels of NO in both serum and tissue, in both cases obtaining higher values in patients compared to the control group. They identified a negative correlation between the serum and tissue levels of NO and the serum and tissue levels of catalase, one of the most important enzymes with antioxidant activity [37]. The investigation of the relationship between nitrosative stress and antioxidant defence was also an objective of our study. Therefore, the aforementioned results are complemented by the findings of our study which revealed lower levels of TAS in CLP patients compared to controls and negative correlations between TAS and both nitrate and nitrite levels in CLP patients, suggesting that the antioxidant capacity in those patients is exceeded.

We also investigated the inflammatory status in CLP patients and the relationship between NO and inflammation. We found significantly higher levels of hsCRP and orosomucoid in CLP patients compared to controls and a positive correlation between serum hsCRP and nitrite levels (direct nitrite and total nitrite). In addition, there was a negative correlation between hsCRP and TAS levels. These results emphasize the close link between the prooxidant–antioxidant balance and inflammation. Alteration of antioxidant defense capacity is associated with a proinflammatory status. Current evidence suggests that the interdependence between oxidative stress and inflammation plays a crucial role; these two processes are simultaneously identified in chronic inflammatory skin conditions [38,39,40].

Similar results to those obtained in our study were also revealed in studies that analyzed nitrosative stress markers in correlation with inflammation in the saliva of patients with oral LP. Shiva et al. showed that there is a direct correlation between the salivary level of NO and the salivary and serum levels of CRP, emphasizing the interconnection between oxidative stress and inflammation. Inflammation induces alterations in lipid metabolisms and chronic inflammation enhances the risk of OLP [41]. Increased salivary NO in OLP patients is assumed to be the result of the release of increased amounts of IL-6, TNF-α or IL1-ß by inflammatory cells such as T cells and macrophages [42]. NO also plays a complex role in cutaneous inflammation. It exhibits pro-inflammatory properties through pathways like the cyclooxygenase/prostaglandin pathway, inflammatory cell migration, and cytokine production. However, NO can also inhibit inflammation by suppressing T cell proliferation and leukocyte migration, promoting T-cell apoptosis, and reducing cytokine production. Topical NO-releasing products show promise in treating conditions like atopic dermatitis in both humans and murine models. The exact mechanisms underlying the dual effects of NO on cutaneous inflammation are not fully understood, needing further investigation [43].

Our study revealed significantly higher serum SDMA levels compared to controls, and we also observed a positive correlation between serum SDMA and hsCRP levels. A negative correlation was identified between SDMA and TAS. These results suggest that SDMA could be considered a nitrosative stress marker in CLP patients and is associated with a pro-inflammatory status. Recent studies have increasingly focused on studying SDMA, a compound that for a long time was considered to be an inert metabolite [44,45]. It is accepted that SDMA is involved in various physiological events in the human body such as inflammation, endothelial dysfunction, oxidative stress, and apoptosis. SDMA increases the expression of differentiation and adhesion markers in monocytes and granulocytes [46]. SDMA does not exert a direct effect on NOS; it acts as an indirect inhibitor of NO, through arginine deficiency. Arginine-N-methyltransferases [PRMTs] are enzymes that contribute to the formation of SDMA. Under conditions of oxidative stress, enzymes involved in SDMA synthesis increase while enzymes responsible for metabolizing the compound decrease [47].

SDMA may promote an inflammatory status, which facilitates the perpetuation of a chronic inflammatory process. In line with this, positive correlations between the serum levels of SDMA and the levels of proinflammatory cytokines such as IL-6 and TNF-alpha have been identified. There is an increase in the synthesis of ROS in association with the inhibition of NO synthesis in endothelial cells when the serum levels of SDMA are elevated. The inhibition of NO synthesis results from the restriction of L-arginine substrate supply to NOS, as SDMA was identified as a strong competitor in transporting L-arginine [48,49].

We also analyzed the influence of disease characteristics and the studied parameters. There were no correlations between nitrite and nitrate levels and disease duration. However, there was a positive correlation between serum nitrate levels and BSA. These results suggest that disease extension directly correlates with NO homeostasis. Regarding the interpretation of these results, it should also be taken into account that only two of the patients had extensive lesions [BSA > 60%].

Our study provides valuable insights into the role of nitrosative stress in the pathogenesis of CLP, as we analyzed the markers related to the NO pathway and their link with both antioxidant capacity and inflammation. Our findings may improve our understanding of NO involvement in the development of LP lesions. However, we should mention that our study included a small number of subjects and was performed in a single centre. Studies on larger groups are necessary to establish the role of the studied markers in medical practice.

## 5. Conclusions

This study found increased serum levels of nitrates, nitrites, and SDMA in CLP patients compared to controls, demonstrating increased nitrosative stress levels among these patients. The low levels of TAS and the negative correlations identified between nitrosative stress markers and TAS suggest an impaired antioxidant defense in CLP patients, and the positive correlations between nitrosative stress markers and CRP indicate the close relationship between inflammation and NO homeostasis. Therefore, analyzed together, these results show that in the pathogenesis of LP, mechanisms related to nitrosative stress and inflammation are interposed.

## Figures and Tables

**Table 1:** Inclusion and exclusion criteria of study participants.

Inclusion Criteria	Exclusion Criteria
otherwise healthy individuals (age over 18 years); individuals with a normal nutritional status; patients diagnosed with cutaneous LP that was confirmed by the histopathological examination; patients diagnosed with cutaneous LP with no previous treatment for the disease.	adults with history for chronic alcohol use, drug abuse; smokers; patients under treatment with drugs that influence the immune response: corticosteroids, immunosuppressant agents (methotrexate, cyclosporine, etc., or nutritional supplements (vitamins, antioxidant compounds, etc.); pregnant and breastfeeding women.

**Table 2:** Serum levels of markers of inflammation and antioxidant defence in CLP patients and controls (expressed as mean and standard deviation).

Parameter	CLP Group (*n* = 40)	Control Group (*n* = 40)	*p* Value
**Inflammatory status**			
hsCRP (mg/dL)	0.55 ± 0.39	0.05 ± 0.02	<0.01
ESR (mm/h)	13.2 ± 12.2	6.2 ± 4.8	0.052
Orosomucoid (g/L)	1.19 ± 0.91	0.72 ± 0.22	0.021
**Antioxidant status**			
TAS (µmol/L)	272.22 ± 41.42	322.57 ± 31.46	<0.01

TAS—total antioxidant status; hsCRP—highly sensitive C-reactive protein.

**Table 3:** Serum levels of nitrosative stress markers in CLP patients and controls (expressed as mean and standard deviation).

Parameter	CLP Group (*n* = 40)	Control Group (*n* = 40)	*p* Value
Direct nitrite (µmol/L)	25.02 ± 4.58	14.85 ± 1.59	<0.01
Total nitrite (µmol/L)	54.9 ± 8.18	33.8 ± 2.23	<0.01
Nitrate (µmol/L)	29.87 ± 4.42	18.95 ± 1.78	<0.01
SDMA (µmol/L)	0.82 ± 0.20	0.49 ± 0.06	<0.01

SDMA—symmetric dimethylarginine.

**Table 4:** The relationship between TAS and nitrosative stress markers in CLP patients.

Parameter	rho	*p*
Direct nitrite (µmol/L)	−0.53	0.02
Total nitrite (µmol/L)	−0.48	0.001
Nitrate (µmol/L)	−0.39	0.013
SDMA (µmol/L)	−0.40	0.031

SDMA—symmetric dimethylarginine; rho—Spearman’s rank correlation coefficient.

**Table 5:** The relationship between hsCRP and nitrosative stress markers and TAS in CLP patients.

Parameter	rho	*p*
Direct nitrite (µmol/L)	0.49	0.001
Total nitrite (µmol/L)	0.39	0.002
Nitrate(µmol/L)	0.26	0.104
SDMA (µmol/L)	0.48	0.001
TAS (µmol/L)	−0.37	0.019

SDMA—symmetric dimethylarginine, TAS—total antioxidant status, rho—Spearman’s rank correlation coefficient.

**Table 6:** The relationship between nitrosative stress markers and clinical characteristics in CLP patients.

Parameter	rho	*p*
**Disease duration**		
Direct nitrite (µmol/L)	0.11	0.402
Total nitrite (µmol/L)	0.09	0.824
Nitrate (µmol/L)	0.16	0.095
SDMA (µmol/L)	0.19	0.219
**Body surface area**		
Direct nitrite (µmol/L)	0.09	0.704
Total nitrite (µmol/L)	0.12	0.172
Nitrate (µmol/L)	0.38	0.013
SDMA (µmol/L)	0.15	0.421

SDMA—symmetric dimethylarginine; rho—Spearman’s rank correlation coefficient.

## Data Availability

All data are contained within the article.
